# Perinatal and Maternal Outcomes According to the Accurate Term Antepartum Ultrasound Estimation of Extreme Fetal Weights

**DOI:** 10.3390/jcm12082995

**Published:** 2023-04-20

**Authors:** Juan Mozas-Moreno, Mariola Sánchez-Fernández, Ernesto González-Mesa, Rocío Olmedo-Requena, Carmen Amezcua-Prieto, José J. Jiménez-Moleón

**Affiliations:** 1 Department of Obstetrics and Gynecology, University of Granada, 18016 Granada, Spain; 2 Obstetrics and Gynecology Service, Virgen de las Nieves University Hospital, 18014 Granada, Spain; 3 Consortium for Biomedical Research in Epidemiology & Public Health (CIBER Epidemiología y Salud Pública-CIBERESP), 28029 Madrid, Spain; 4 Biohealth Research Institute (Instituto de Investigación Biosanitaria Ibs.GRANADA), 18014 Granada, Spain; 5 Obstetrics and Gynecology Service, La Inmaculada Hospital, Huércal-Overa, 04600 Almería, Spain; 6 Instituto de Investigación Biomédica de Málaga y Plataforma en Nanomedicina (IBIMA-Plataforma BIONAND), Research Group in Maternal-Fetal Medicine, Epigenetics, Women’s Diseases and Reproductive Health, 29071 Málaga, Spain; 7 Obstetrics and Gynecology Service, Regional University Hospital of Malaga, 29011 Malaga, Spain; 8 Department of Surgical Specialties, Biochemistry and Immunology, University of Malaga, 29071 Malaga, Spain; 9 Department of Preventive Medicine and Public Health, Faculty of Medicine, University of Granada, 18016 Granada, Spain

**Keywords:** fetal growth, fetal weight, sonographic estimated fetal weight, accuracy, fetal macrosomia, fetal microsomia, large for gestational age, small for gestational age

## Abstract

(1) Background: The accuracy of ultrasound estimation of fetal weight (EFW) at term may be useful in addressing obstetric complications since birth weight (BW) is a parameter that represents an important prognostic factor for perinatal and maternal morbidity. (2) Methods: In a retrospective cohort study of 2156 women with a singleton pregnancy, it is verified whether or not perinatal and maternal morbidity differs between extreme BWs estimated at term by ultrasound within the seven days prior to birth with Accurate EFW (difference < 10% between EFW and BW) and those with Non-Accurate EFW (difference ≥ 10% between EFW and BW). (3) Results: Significantly worse perinatal outcomes (according to different variables such as higher rate of arterial pH at birth < 7.20, higher rate of 1-min Apgar < 7, higher rate of 5-min Apgar < 7, higher grade of neonatal resuscitation and need for admission to the neonatal care unit) were found for extreme BW estimated by antepartum ultrasounds with Non-Accurate EFW compared with those with Accurate EFW. This was the case when extreme BWs were compared according to percentile distribution by sex and gestational age following the national reference growth charts (small for gestational age and large for gestational age), and when they were compared according to weight range (low birth weight and high birth weight). (4) Conclusions: Clinicians should make a greater effort when performing EFW by ultrasound at term in cases of suspected extreme fetal weights, and need to take an increasingly prudent approach to its management.

## 1. Introduction

Birth weight (BW) is a parameter that represents an important prognostic factor for perinatal and maternal morbidity. Low BW (microsomia) is one of the major determinants of perinatal mortality and morbidity, associated with low Apgar test scores, low arterial umbilical cord blood pH, and a higher rate of admission to the neonatal care unit, with an increase in mother-infant separation [1]. Likewise, macrosomic fetuses (high BW) are at greater risk of suffering from shoulder dystocia during delivery and the associated morbidities such as brachial palsy, facial paralysis, neurological alterations, and bone fractures. Moreover, maternal complications associated with fetal macrosomia include an increased risk of cesarean section, instrumental deliveries, traumas of the birth canal, and severe perineal tears, with a consequent delay in postpartum maternal recovery [2].

An obstetric ultrasound is considered a routine test used throughout pregnancy to evaluate gestational age, fetal morphology, and fetal growth, with the latter usually using estimated fetal weight (EFW) [3]. Thus, the evaluation of fetal growth is presented as a critical component of prenatal care, which allows for the identification of cases with a higher risk of maternal and perinatal morbidity, such as fetuses with extreme weights. The accuracy of EFW by ultrasound has improved with current ultrasound equipment, although the lack of complete success is still evident [4]. Certain maternal, obstetric, and ultrasound performance variables have been considered potential determinants of EFW accuracy. These factors include ethnic differences, maternal obesity, amniotic fluid volume, sex of the fetus, fetal presentation, location of the placenta, time interval between ultrasound and delivery, the role of the observers, and cases of extreme fetal weights [5].

The systematic practice of ultrasounds regarding at term pregnancies is generally questioned [6,7]. However, the poor maternal and perinatal outcomes of fetuses with extreme weights could favor their use in identifying fetuses with extreme weight and modifying antepartum and intrapartum care in these cases in order to prevent maternal and perinatal complications [2,8,9].

On the other hand, the question of whether maternal and perinatal outcomes vary in the success of the routine ultrasonic diagnosis of EFW at term remains unresolved. In this sense, there are barely any studies that evaluate the maternal and perinatal outcomes following antepartum EFW with a focus on extreme fetal weights, assessing the implications of errors in the calculation of the EFW at term for maternal and perinatal outcomes [10].

Thus, the objective of this study was to evaluate birth outcomes to verify whether perinatal and maternal morbidity (related to childbirth) differ between Accurate EFW (difference < 10% between EFW on ultrasound at term and BW) and Non-Accurate EFW (difference ≥ 10% between EFW at term ultrasound and BW) of the different categories of extreme BWs diagnosed antepartum within seven days prior to birth, according to percentile distribution (SGA, LGA) or range distribution (macrosomia or microsomia).

## 2. Materials and Methods

### 2.1. Study Design, Setting, and Participants

This is a single-center retrospective cohort observational study that reviewed the last 2500 clinical histories of pregnant women who gave birth during the period 2017–2019 at the public hospital La Inmaculada, Huércal-Overa, Almería, Spain, where 1300 births are attended annually with no obstetrics teaching activity. The sample was selected from the target population and included all pregnant women who fulfilled the following inclusion criteria: singleton pregnancy, full-term delivery certified through a first-trimester ultrasound, fetal biometry conducted at a prenatal visit within seven days prior to giving birth showing an intact amniotic sac; they also did not present any of the exclusion criteria: multiple pregnancies, pre-ultrasound rupture of the amniotic sac, pre-term or post-term delivery (i.e. <37 or >42 weeks of gestation), fetal death, fetal transverse static, fetal malformation, uterine malformation, or a time period of more than seven days between the last fetal biometry and delivery. The final sample consisted of 2156 pregnant women, who did not present any of the exclusion criteria and from whom the pertinent information was collected. 

### 2.2. Data Sources and Variables

BW was used as the final test at birth for the accuracy of the ultrasound calculation of the intrauterine EFW and was measured using the same weighing scale, which was located in the hospital delivery room or in the neonatal care unit when the neonate required urgent admission. The BWs were classified as either low weight (<2500 g; microsomia), high weight (>4000 g; macrosomia), or normal weight (2500–4000 g), regardless of gestational age. In addition, using BW, the newborns were also classified according to gestational age, with small for gestational age (SGA) being under the 10th percentile, large for gestational age (LGA) being above the 90th percentile, and appropriate for gestational age (AGA) being between the 10th and 90th percentile, following the percentile distribution of the national reference growth charts according to sex, singleton pregnancy, and gestational age, following the most internationally accepted definitions [11]. 

EFWs at term were routinely determined in the hospital following the local protocol and each calculation was made with the same ultrasound equipment (Toshiba Medical System Xario SSA-660A; Otawara, Tochigi, Japan) operated by one of the eight staff gynecologists with over six years of experience in fetal biometrics and who attended the prenatal appointments on a rotational basis. The staff was not blinded to the results as the data were collected as part of routine clinical practice. To measure the bi-parietal diameter (BPD) a median transaxial plane was taken at the point where the midline was interrupted by the septum pellucidum and the thalami. Abdominal circumference (AC) was measured in the plane that passes at the level of the liver, looking at the fetal portal system and with a perpendicular cut of the rachis. This circumference was estimated indirectly, that is, with the antero-posterior and transverse diameters of the abdomen. The calipers were placed on the outer table of the parietals for the BPD and on the fetal skin for the AC. Femur length (FL) was measured along the major axis of the diaphysis, avoiding curvature from the greater trochanter to the lateral condyle, while avoiding the head of the femur and the distal epiphysis. The formula used to calculate the EFW was that proposed by Hadlock et al. [12] (Hadlock 2): Log10 EW = 1.335 − (0.0034 × AC × FL) + (0.0316 × BPD) + (0.0457 × AC) + (0.1623 × FL).

Gestational age was calculated according to the last menstrual period and was corrected when there was a discrepancy of more than seven days between this and the date established by the first-trimester ultrasound (based on the cranio-caudal length). The location of the placenta was classified as anterior when the insertion of the placenta was anterior or fundal, whereas it was considered posterior when the insertion of the placenta was totally posterior. The quantity of amniotic fluid was estimated according to the four quadrants technique developed by Phelan et al. [13] in which it was classified as normal when the amniotic fluid index (AFI) was between 5 cm and 21 cm, scarce when it was <5 cm, and abundant when it was >21 cm, and dichotomized into normal vs. scarce or abundant (oligo or polihydramnios) for a comparison of the baseline characteristics of the groups. Likewise, the days elapsed between the ultrasound and delivery were divided into 0–4 vs. 5–7 days (maximum seven days) to allow comparison of the baseline characteristics of the groups.

Maternal BMI was calculated using the maternal weight and height measurements obtained at the first antenatal appointment according to the formula BMI = weight (kg)/height^2^ (m^2^). BMI categories were defined as follows: normal weight (BMI 18.5–24.9 kg/m^2^), overweight (BMI 25–29.9 kg/m^2^), obese (BMI > 29.9 kg/m^2^), and underweight (BMI < 18.5 kg/m^2^); for comparison purposes they were summarized as <25 vs. >25 kg/m^2^. Maternal ethnicity was also grouped into Caucasian vs. Others. Maternal pathology associated with pregnancy was also grouped into None vs. Others. 

The perinatal outcome variables considered were: type of delivery (spontaneous vaginal delivery vs. instrumental vaginal delivery or cesarean section); perineal tear (none or skin and vaginal mucosa – 1st type- vs. higher grade -2nd, 3rd or 4th type- and/or need for episiotomy); shoulder dystocia (failure to deliver the fetal shoulders using solely gentle downward traction or requirement of additional delivery maneuvers are needed to successfully deliver the baby) or trauma obstetric with skin; nerve or bone involvement (no vs. yes); type of neonatal resuscitation (none or aspiration of secretions -1st type- vs. higher grade); need for newborn to be admitted to the neonatal care unit (no vs. yes); Apgar test score at one minute and at five minutes (7 to 10 vs. <7); and arterial pH at birth (>7.20 vs. <7.20).

### 2.3. Statistical Analysis

In the descriptive analysis of the sample, means (SD) were calculated for the continuous quantitative variables while absolute and relative frequencies were presented for the qualitative variables. For each category of extreme fetal weights at birth, newborns who had an EFW error <10% of the BW (estimates that fell within the intervals {0.90 × BW, 1.10 × BW}) were considered Accurate EFW and those who had a difference ≥10% between EFW at term ultrasound and BW were considered Non-Accurate EFW. Student’s *t*-test was used to compare the mean maternal age (SD) of the study groups, while Chi square and Fisher’s exact tests were used for categorical variables, as appropriate. The level of significance considered for all analyses was *p* < 0.05.

## 3. Results

The total sample of newborns who had undergone antepartum EFW was 2156. The percentages of BW according to percentile distribution by sex and gestational age following the national reference growth charts were SGA 7.70%, LGA 12.99%, and AGA 79.31%, and according to weight range were low BW (microsomia) 3.01%, high BW (macrosomia) 7.56%, and normal BW 89.42%. 

Of the entire sample, there were 1621 (75.20%) cases in the EFW Accurate group and 535 (24.80%) cases in the EFW Non-Accurate group, with a significant increase in cases in the EFW Non-Accurate group for extreme BW according to percentile distribution and BW range (*p* < 0.001 in both cases) (Table 1).

The mean age of the pregnant women was 29.54 (±5.84) years, with no significant difference between the EFW Accurate and EFW Non-Accurate study groups (*p* = 0.664). Maternal ethnicity was Caucasian 71.15% and Others 28.85% (Arab 11.51%, South American 10.34%, Gypsy 5.84%, or African American 1.16%). Maternal pathology associated with pregnancy was None 88.13% and Others 11.78% (gestational diabetes 9.88%, pregestational diabetes 0.32%, gestational hypertension or preeclampsia 1.48%, or pregestational hypertension 0.19%). Other baseline characteristics of the study groups are presented in Table 2. No significant differences were found between the Accurate and Non-Accurate EFW groups, except for the parity variable, in which there were higher rates of nulliparous pregnancies in the Non-accurate EFW group (*p* = 0.014).

The mode of delivery globally was 1631 (75.61%) spontaneous vaginal, 171 (7.93%) operative vaginal, and 218 (16.46%) had a cesarean section. In total, 137 (6.35%) pre-labor cesarean sections and 218 (10.11%) during-labor cesarean sections were performed. No differences in adverse maternal outcomes were found among those studied (mode of delivery or perineal tear) for any of the extreme BW categories compared according to percentile distribution by sex and gestational age following the national reference growth charts or according to weight range. Moreover, no significant group differences were found for shoulder dystocia or fetal trauma.

Nevertheless, when the antepartum EFW of extreme BW according to percentile distribution by sex and gestational age following the national reference growth charts were compared for SGA newborns it was found that, among the perinatal outcome variables studied, newborn cases with Non-Accurate EFW required a higher grade of neonatal resuscitation (2nd, 3rd or 4th type) (*p* = 0.025) and had a higher rate of arterial pH at birth < 7.20 (*p* = 0.001) than those with Accurate EFW (Table 3). 

For the LGA newborns, it was also found that those with Non-Accurate EFW required a higher degree of neonatal resuscitation (2nd, 3rd or 4th type) (*p* = 0.025), greater need for admission to the neonatal care unit (*p* < 0.001), and had a higher rate of arterial pH at birth <7.20 (*p* = 0.002) than those with Accurate EFW (Table 4).

When comparing the antepartum EFW of extreme BW according to weight range, for microsoma newborns it was found that, among the maternal and perinatal outcome variables studied, newborn cases with Non-Accurate EFW required higher grade neonatal resuscitation (2nd, 3rd, or 4th type) (*p* = 0.009), had a higher rate of 1-min Apgar < 7 (*p* = 0.044), higher rate of 5-min Apgar < 7 (*p* = 0.005), and a higher rate of Arterial pH at birth < 7.20 (*p* = 0.010) than those with Accurate EFW (Table 5). 

For the macrosoma newborns, it was also found that those with Non-Accurate EFW required a higher degree of neonatal resuscitation (2nd, 3rd, or 4th type) (*p* = 0.010), and had a greater need for admission to the neonatal care unit (*p* < 0.001) than those with Accurate EFW (Table 6). 

## 4. Discussion

Extreme BW is a well-documented factor involved in adverse maternal and perinatal outcomes [14]. The current study found that perinatal morbidity differs between extreme BW diagnosed by antepartum term ultrasound within seven days prior to birth, that is, between Accurate EFWs (difference < 10% between ultrasound EFW and BW) and Non-Accurate EFWs (difference ≥ 10% between ultrasound EFW and BW). Overall, worse perinatal outcomes were found for newborns with Non-Accurate EFW than those with Accurate EFW. This was the case when extreme BWs were compared according to percentile distribution by sex and gestational age following the national reference growth charts, with a higher degree of neonatal resuscitation (2nd, 3rd, or 4th type) and a higher rate of arterial pH at birth < 7.20 for Non-Accurate SGA and LGA, as well as a greater need for admission to the neonatal care unit for Non-Accurate LGA. Likewise, it was also confirmed that when extreme BWs were compared according to weight range, a higher degree of neonatal resuscitation (2nd, 3rd, or 4th type), higher rates of Arterial pH at birth (<7.20), and higher rates of 1-min Apgar (<7) and 5-min Apgar (<7) were observed for Non-accurate microsoma (low BW); we observed a higher degree of neonatal resuscitation (2nd, 3rd, or 4th type) and a greater need for admission to the neonatal care unit in Non-Accurate macrosoma (high BW). The threshold for categorizing Accurate and Non-accurate EFW was set at ±10% since an estimate of fetal weight with more than ±10% variation is likely to affect decision-making in clinical practice [4,5,15].

The comparison groups were homogeneous, since there was no significant difference between the Accurate and Non-Accurate EFW groups, except for the parity variable, in which nulliparous pregnant women had a higher rate of Non-Accurate EFW. This could be explained by the fact that clinicians tend to be more meticulous when performing ultrasounds in multiparous pregnancies since these women are older and have a higher risk of arterial hypertension, gestational diabetes, macrosomic fetuses, shoulder dystocia, and severe perineal tears. Likewise, in our study, the high prevalence of LGA (12.99%) and macrosomia (7.56%) could be explained by the high prevalence of gestational diabetes (9.88%) in the subject population. This, in turn, is probably due to the high prevalence of overweight and obese women (BMI > 25 Kg/m^2^) in the study sample (45.18%). In contrast, the prevalence of microsomia (3.01%) is low, which is most likely due to the fact that pregnant women with preterm labor and gestational age < 33 weeks were transferred to a referral hospital for preterm births prior to delivery. 

Mathematical formulas for EFW have been shown to have large errors at the extremes of a BW range [5]. The use of Hadlock’s formula for EFW, which was considered in this study, since it was used systematically for the antepartum EFW at the hospital, and commonly used in our field, has been supported by large groups, such as that of Nicolaides et al. [16] who, following a review of the literature, confirmed its superiority in the prediction of EFW [17].

Many different factors during pregnancy, labor, and delivery can affect cord blood gases and Apgar score at birth [18]. Chronic antepartum fetal hypoxia due to maternal or placental causes is another mechanism that results in fetal growth restriction and perinatal mortality or serious neonatal morbidity. The morbidity associated with excessive fetal growth includes polycythemia, hyperbilirubinemia, and hypoglycemia, which occurs secondarily to fetal hyperinsulinemia and consequent fetal hypoxia [11]. Thus, alterations in arterial pH and in Apgar test scores at birth occur more frequently in cases of extreme fetal weights. In our study, we found an increase in these alterations in the extreme fetal weights with Non-Accurate EFW compared to Accurate EFW. Taking into account that this assessment of the EFW was known by the clinicians who attended the deliveries, it may be speculated that there were undetected differences in the fetal reserve of extreme fetal weights with Non-Accurate EFW that would make these fetuses more susceptible to intrapartum distress.

Fetal growth abnormalities are commonly diagnosed using criteria such as low BW (microsomia), high BW (macrosomia), SGA, and LGA. Numerous risk factors for fetal growth restriction and excessive fetal growth have been identified and can be classified into maternal, fetal, and placental factors. Abnormalities of fetal growth serve as indicators of pregnancy complications and are associated with adverse perinatal outcomes. Intrauterine growth restriction defines diminished growth velocity in the fetus occurring in utero, as documented by at least two intrauterine growth measurements, and the fetus does not reach the genetically determined growth potential. The term SGA, on the other hand, refers to the size of the infant at birth according to gestational age and gender, compared to reference data. Not all intra-uterine growth restriction babies are born SGA and not all SGA-born babies are intra-uterine growth restriction. However, in studies, these terms have been used interchangeably. Growth vulnerabilities assessed antenatally (intrauterine growth restriction) and at the time of birth (SGA) are significantly associated with an increased risk of perinatal morbidity, and also a greater susceptibility to developing diseases (especially cardio-metabolic and neurological disorders) later in life. Emerging evidence has supported the hypothesis of the Developmental Origin of Health and Disease, which states that individual developmental “programming” takes place via a delicate fine-tuning of fetal genetic and epigenetic marks in response to a large variety of “stressor” exposures during pregnancy [19,20,21].

We adopted this dual approach to extreme BW (compared according to percentile distribution by sex and gestational age following the national reference growth charts and according to weight range) due to the fact that the perinatal literature includes several potentially confusing and controversial terms and concepts related to fetal-neonatal size and growth. Although the classification of extreme BW into SGA and LGA is more specific, microsomia (<2500 g) and macrosomia (≥4000 g) are indices of size with substantial utility for various reasons, including ease of measurement and strong correlation with adverse perinatal outcomes [11]. However, ideally, we would move from a percentile-based identification of fetal growth restriction and excess growth to a system based on a clinically relevant outcome-based criterion [22].

Krispin et al. [10] conducted a case-control study of pregnancies that underwent fetal biometry seven days before delivery using the Hadlock formula, where they compared two groups according to the differences between EFW and BW, with a difference of ±20% (Non-accurate EFW) and ±1% (Accurate EFW), in an attempt to establish an association between the inaccuracy of 2D ultrasounds and subsequent perinatal implications. Their findings were similar to those reported in our study. Sonographic inaccuracy was not related to different maternal outcomes (i.e., labor induction rates, mode of delivery, use of regional analgesia, post-partum hemorrhage, episiotomy, or 3rd or 4th-degree perineal tear), or with the presentation of shoulder dystocia. However, a significant association was found between such an inaccuracy and adverse neonatal outcomes, such as respiratory distress, composite respiratory outcomes (including transient tachypnea of the newborn, respiratory distress syndrome, mechanical ventilation, and meconium aspiration), and neonatal intensive care unit admissions. However, in that study, a comparison was made between very extreme EFW diagnostic groups, one with almost complete accuracy (±1%) versus another with a much wider error margin (±20%) than that used in our study. In our study, the percentage of EFWs with an error of less than 10% of the BW was calculated, that is, in the range of 0.90 × BW to 1.10 × BW (Accuracy ratio), following the methodology employed by other studies considering the accuracy of EFW [17,23,24,25,26].

Despite the limitations in its accuracy, fetal biometry with EFW calculation remains the current gold standard for the diagnosis of abnormal fetal growth, since none of the newly proposed alternatives (such as three-dimensional ultrasound and magnetic resonance imaging) have clearly shown to be superior [11]. Ultrasonography is used selectively in late pregnancy when there are specific clinical indications. However, the value of routine ultrasound screening late in pregnancy in unselected populations is controversial. The rationale for such screening would be the detection of clinical conditions that put the fetus or mother at high risk, which would not necessarily have been detected by other means, such as clinical examination, and whose subsequent management would improve perinatal outcomes. Based on existing evidence, routine ultrasounds in late pregnancy in low-risk or unselected populations do not appear to be beneficial for the mother or baby and may be associated with a small increase in caesarean section rates. There is a lack of data on the possible psychological effects of routine ultrasounds in late pregnancy and limited data on its effects on short- and long-term neonatal and child outcomes [7].

The World Health Organization recommends that no routine ultrasound be performed after the 24th week of gestation and, in any case, should only be carried out with the intention of identifying the number of fetuses, fetal presentation, and location of the placenta [6]. A Cochrane review [7] concluded that, based on the evidence analyzed, and assuming the lack of information on long-term neurodevelopment, routine ultrasonography at the end of pregnancy in low-risk or unselected populations does not provide benefits to either the mother or the fetus, finding no differences in perinatal morbidity and mortality, preterm delivery before 37 weeks, caesarean section rates, or labor induction. In addition, results from other studies warn of the possible negative consequences of implementing ultrasounds, particularly those related to unnecessary medical interventions or the induction of maternal anxiety [27], and some even explicitly advise against this procedure, stating that it is not only a poor tool for predicting restricted intra-uterine growth, but it can be potentially dangerous, since it leads to an increase in labor induction and cesarean section rates, without having demonstrated an improvement in neonatal prognosis [28].

However, although the definitive category that an infant has only will be known after delivery, the excellent safety profile, ease of use, and wide availability have made ultrasound a modality of choice for intrauterine fetal evaluation, and, for many clinicians, antepartum evaluation. Likewise, it must be considered that clinicians often take ultrasound-based EFW as an indicator of BW. This is usually done without due consideration of factors that may affect its accuracy, such as extreme fetal weights [5]. This is precarious, since an inaccurate EFW may lead to unnecessary or delayed interventions, putting both mother and fetus at risk [15]. On the other hand, the validity of ultrasound EFW is influenced by extreme fetal weights, with a tendency to overestimate low weights and underestimate high weights, which represents a clinically important finding [29]; the inaccuracy of the EFW in extreme fetal weights at term is associated with worse perinatal outcomes [10], as also suggested by the results of our study. Thus, since the accuracy of ultrasound EFW was reduced according to the observers and in extreme fetal weights [30], greater care should be taken on the part of clinicians when performing EFW by ultrasound at term in cases of suspected extreme fetal weights. In particular, efforts must be made to minimize the variability of EFW if it is to be clinically useful. This may be achieved through making several measurements of each ultrasound parameter when performing the EFW and averaging the result, improving image quality, ensuring uniform calibration of equipment, carefully designing and refining measurement methods, acknowledging that there is a long learning curve, and regular auditing of measurement quality. Further work is also required to improve the universal validity and accuracy of EFW formulae [5].

The main limitations of this study concern the retrospective design and the lack of randomization of the pregnant women (according to whether the pregnancy was normal or pathological) with regard to the observers. Nevertheless, since the observers were assigned to the consultations in a rotating order, we might argue that spontaneous randomization has been employed. In addition, analysis of data obtained from a single physician center with ultrasound examinations conducted only by experienced sonographers allowed us to minimize possible non-objective measurements that could serve as confounding variables. Likewise, the perinatal mortality variable was not used, although this variable is more difficult to compare due to its infrequency, and in any case, fetal death was an exclusion criterion in our study due to the alteration in the calculation of the EFW. Some baseline characteristics of the population studied, such as extreme birth weight rates or pathology associated with pregnancy, may be different from other populations, so the external validity of the study may be affected. A strength of our study is the high number of cases analyzed and the inclusion of a multi-ethnic population that more closely resembles the current reality of globalized clinical practice.

## 5. Conclusions

Worse perinatal outcomes (as indicated by variables such as higher rate of Arterial pH at birth < 7.20, higher rate of 1-min Apgar < 7, higher rate of 5-min Apgar < 7, higher grade of neonatal resuscitation, and need for admission to the neonatal care unit) were found for extreme BW diagnosed by antepartum term ultrasounds conducted within seven days prior to birth, with Non-Accurate EFW (difference ≥ 10% between EFW ultrasound and BW) compared to those with Accurate EFW (difference < 10% between EFW ultrasound and BW). This result was found when extreme BWs were compared according to percentile distribution by sex and gestational age following the national reference growth charts (SGA and LGA) and was also verified when extreme BWs were compared according to weight range (low BW—microsoma and high BW—macrosoma). Consequently, clinicians should take greater care when performing EFW by ultrasound at term in cases of suspected extreme fetal weights, while a more prudent approach should also be adopted in the management of such cases. 

## Figures and Tables

**Table 1:** Study groups according to percentile distribution and range distribution of birth weights.

Group	Accurate EFW	Non-Accurate EFW	*p* Value
	*n* (%)	*n* (%)	
**Percentile distribution. *n* (%)**			**<0.001**
AGA. 1710 (79.31)	1343 (78.50)	367 (21.50)	
SGA. 166 (7.70)	86 (51.80)	80 (48.20)	
LGA. 280 (12.99)	192 (68.60)	88 (31.40)	
**Range distribution. *n* (%)**			**<0.001**
Normal BW. 1928 (89.42)	1488 (77.18)	440 (22.82)	
Microsoma. 65 (3.01)	33 (50.77)	32 (49.23)	
Macrosoma. 163 (7.56)	100 (61.35)	63 (38.65)	
**Total *n* (%)**			
2156 (100)	1621 (75.18)	535 (24.82)	

EFW: estimated fetal weight; BW: birth weight; SGA: Small for gestational age (<10th percentile according to national reference growth charts); LGA: Large for gestational age (>90th percentile according to national reference growth charts); AGA: Appropriate for gestational age (between 10th and 90th per-centile according to national reference growth charts); Normal BW (between 2500 and 4000 g); Macrosoma: high BW (>4000 g); Microsoma: low BW (<2500 g).

**Table 2:** Baseline characteristics of the study groups.

Group	Accurate EFW 1621 (75.18)	Non-Accurate EFW 535 (24.82)	*p* Value
Variable	*n* (%)	*n* (%)	
**Fetal sex**			
Male	848 (75.71)	272 (24.29)	0.555
Female	773 (74.61)	263 (25.39)	
**Fetal presentation**			
Vertex	1558 (75.19)	514 (25.12)	0.998
Breech	63 (75.00)	21 (25.00)	
**Placental location**			
Anterior	945 (74.88)	317 (25.12)	0.697
Posterior	676 (75.61)	218 (24.39)	
**Amniotic fluid**			
Normal	1510 (75.19)	514 (24.81)	0.613
Oligo or polihydramnios	111 (75.00)	37 (25.00)	
**Delivery gestation weeks**			
37–39	717 (76.80)	217 (23.20)	0.137
40–41	904 (74.00)	318 (26.00)	
**Body mass index (kg/m^2^)**			
<25	895 (75.70)	287 (24.30)	0.527
>25	726 (74.54)	248 (25.46)	
**Parity**			
0	734 (72.74)	275 (27.26)	**0.014**
>1	887 (77.33)	260 (22.67)	
**Ethnicity**			
Caucasian	1144 (74.60)	390 (25.40)	0.304
Others	477 (76.70)	145 (23.30)	
**Days from ultrasound to birth**			
0–4	1217 (75.10)	403 (24.90)	0.908
5–7	404 (75.40)	132 (24.60)	
**Pathology associated with pregnancy**			
None	1428 (75.20)	472 (24.80)	0.935
Others	193 (75.40)	63 (24.60)	

EFW: estimated fetal weight; Other ethnicities: Arab (11.51%), South American (10.34%), Gypsy (5.84%), African American (1.16%); Others pathologies associated with pregnancy: gestational diabetes (9.88%), pregestational diabetes (0.32%), gestational hypertension or preeclampsia (1.48%), pregestational hypertension (0.19%).

**Table 3:** Small for gestational age newborns: labor, maternal, and perinatal outcomes according to the Accurate or Non-Accurate EFW.

Group	Accurate EFW	Non-Accurate EFW	*p* Value
Variable	*n* (%)	*n* (%)	
**Mode of delivery**			
Spontaneous vaginal	64 (53.80)	55 (46.20)	0.418
Operative vaginal or cesarean	22 (46.20)	25 (53.20)	
**Perineal tear**			
No or 1st type	52 (56.50)	40 (43.50)	0.216
2nd, 3rd, or 4th type, and/or episiotomy	15 (44.10)	19 (55.90)	
**Shoulder distocia or fetal trauma**			
No	64 (52.90)	57 (47.10)	0.998
Yes	3 (60.00)	2 (40.00)	
**Neonatal resuscitation**			
No or 1st type	76 (55.90)	60 (44.10)	**0.025**
2nd, 3rd, or 4th type	10 (33.30)	20 (66.70)	
**Neonatal care unit**			
No	63 (55.30)	51 (44.70)	0.124
Yes	23 (44.20)	29 (55.80)	
**1-min Apgar**			
7–10	77 (53.80)	66 (46.20)	0.190
<7	9 (39.10)	14 (60.90)	
**5-min Apgar**			
7–10	80 (54.10)	68 (45.90)	0.097
<7	6 (33.30)	12 (66.70)	
**Arterial pH at birth**			
>7.20	71 (57.70)	52 (42.30)	**0.001**
<7.20	7 (23.30)	23 (76.70)	

EFW: estimated fetal weight; Small for gestational age newborns: <10th percentile according to national reference growth charts.

**Table 4:** Large for gestational age newborns: labor, maternal, and perinatal outcomes according to the Accurate or Non-Accurate EFW.

Group	Accurate EFW	Non-Accurate EFW	*p* Value
Variable	*n* (%)	*n* (%)	
**Mode of delivery**			
Spontaneous vaginal	135 (68.90)	61 (31.10)	0.866
Operative vaginal or cesarean	57 (67.90)	27 (32.10)	
**Perineal tear**			
No or 1st type	86 (68.80)	39 (31.20)	0.998
2nd, 3rd, or 4th type/and or episiotomy	64 (68.80)	29 (31.20)	
**Shoulder distocia or fetal trauma**			
No	126 (70.80)	52 (29.20)	0.183
Yes	24(60.00)	16 (40.00)	
**Neonatal resuscitation**			
No or 1st type	175 (70.90)	72 (29.10)	**0.025**
2nd, 3rd, or 4th type	17 (51,50)	16 (48.50)	
**Neonatal care unit**			
No	168 (75.70)	54 (24.30)	**<0.001**
Yes	24 (41.40)	34 (58.60)	
**1-min Apgar**			
7–10	172 (69.10)	77 (30.90)	0.606
<7	20 (64.50)	11 (35.50)	
**5-min Apgar**			
7–10	185 (69.00)	83 (31.00)	0.435
<7	7 (58.30)	5 (41.70)	
**Arterial pH at birth**			
>7.20	167 (72.30)	63 (27.70)	**0.002**
<7.20	15 (45.50)	18 (54.50)	

EFW: estimated fetal weight; Large for gestational age newborns: >90th percentile according to national reference growth charts.

**Table 5:** Microsoma newborns: labor, maternal and perinatal outcomes according to the Accurate or Non-Accurate EFW.

Group	Accurate EFW	Non-Accurate EFW	*p* Value
Variable	*n* (%)	*n* (%)	
**Mode of delivery**			
Spontaneous vaginal	22 (53.70)	19 (46.30)	0.543
Operative vaginal or cesarean	11 (45.80)	13 (54.20)	
**Perineal tear**			
No or 1st type	18 (56.20)	14 (43.80)	0.538
2nd, 3rd, or 4th type/and or episiotomy	6 (46.20)	19 (55.90)	
**Shoulder distocia or fetal trauma**			
No	24 (53.30)	21 (46.70)	-
Yes	0 (0.00)	0 (0.00)	
**Neonatal resuscitation**			
No or 1st type	31 (58.50)	22 (41.50)	**0.009**
2nd, 3rd, or 4th type	2 (16.70)	10 (83.30)	
**Neonatal care unit**			
No	17 (63.00)	10 (37.00)	0.097
Yes	16 (42.10)	22 (57.90)	
**1-min Apgar**			
7–10	31 (56.40)	24 (43.60)	**0.044**
<7	2 (20.00)	8 (80.00)	
**5-min Apgar**			
7–10	33 (56.90)	25 (43.10)	**0.005**
<7	0 (0.00)	7 (100.00)	
**Arterial pH at birth**			
>7.20	27 (58.70)	19 (41.30)	**0.010**
<7.20	2 (16.70)	10 (83.30)	

EFW: estimated fetal weight; Microsoma: low birth weight (<2500 g).

**Table 6:** Macrosoma newborns: labor, maternal and perinatal outcomes according to the Accurate or Non-Accurate EFW.

**Group**	**Accurate EFW**	**Non-Accurate EFW**	***p* Value**
**Variable**	***n* (%)**	***n* (%)**	
**Mode of delivery**			
Spontaneous vaginal	71 (62.80)	43 (37.20)	0.559
Operative vaginal or cesarean	29 (58.00)	21 (42.00)	
**Perineal tear**			
No or 1st type	45 (64.30)	25 (35.70)	0.324
2nd, 3rd, or 4th type/and or episiotomy	30 (55.60)	24 (44.40)	
**Shoulder distocia or fetal trauma**			
No	58 (63.70)	33 (36.30)	0.219
Yes	17 (51.50)	16 (48.50)	
**Neonatal resuscitation**			
No or 1st type	92 (65.20)	49 (34.80)	**0.010**
2nd, 3rd, or 4th type	8 (36.40)	14 (63.60)	
**Neonatal care unit**			
No	83 (70.30)	35 (29.70)	**<0.001**
Yes	17 (37.80)	89 (62.20)	
**1-min Apgar**			
7–10	91 (62.80)	54 (37.20)	0.294
<7	9 (50.00)	9 (50.00)	
**5-min Apgar**			
7–10	95 (65.40)	59 (38.30)	0.735
<7	5 (55.60)	4 (44.40)	
**Arterial pH at birth**			
>7.20	85 (65.40)	45 (34.60)	0.058
<7.20	11 (45.80)	13 (54.20)	

EFW: estimated fetal weight; Macrosoma: high birth weight (>4000 g).

## Data Availability

No new data were created or analyzed in this study. Data sharing is not applicable to this article.
